# Resonant multiphoton processes and excitation limits to structural dynamics

**DOI:** 10.1063/4.0000239

**Published:** 2024-03-01

**Authors:** William J. C. Francis, Harmanjot Grewal, Alexander A. C. Wainwright, Xuchun Yang, Massimo Olivucci, R. J. Dwayne Miller

**Affiliations:** 1 Department of Chemistry, University of Toronto, 80 St. George Street, Toronto, Ontario M5S 3H6, Canada and Department of Physics, University of Toronto, 60 St. George Street, Toronto, Ontario M5S 3J1, Canada; 2 Department of Chemistry, Bowling Green State University, Overman Hall, Bowling Green, Ohio 43403, USA; 3 Department of Biotechnology, Chemistry and Pharmacy, University of Siena, via Aldo Moro 2, Siena I-53100, Italy

## Abstract

Understanding the chemical reactions that give rise to functional biological systems is at the core of structural biology. As techniques are developed to study the chemical reactions that drive biological processes, it must be ensured that the reaction occurring is indeed a biologically relevant pathway. There is mounting evidence indicating that there has been a propagation of systematic error in the study of photoactive biological processes; the optical methods used to probe the structural dynamics of light activated protein functions have failed to ensure that the photoexcitation prepares a well-defined initial state relevant to the biological process of interest. Photoexcitation in nature occurs in the linear (one-photon per chromophore) regime; however, the extreme excitation conditions used experimentally give rise to biologically irrelevant multiphoton absorption. To evaluate and ensure the biological relevance of past and future experiments, a theoretical framework has been developed to determine the excitation conditions, which lead to resonant multiphoton absorption (RMPA) and thus define the excitation limit in general for the study of structural dynamics within the 1-photon excitation regime. Here, we apply the theoretical model to bacteriorhodopsin (bR) and show that RMPA occurs when excitation conditions exceed the linear saturation threshold, well below typical excitation conditions used in this class of experiments. This work provides the guidelines to ensure excitation in the linear 1-photon regime is relevant to biological and chemical processes.

## INTRODUCTION

The use of time-resolved serial femtosecond crystallography (TR-SFX) allows the structural dynamics of proteins to be observed on the femtosecond timescale.^1–3^ After photoexcitation with an ultrafast laser source, the reaction is initiated, and intermediate protein structures are captured as conformational changes occur. The intermediate structures observed by TR-SFX only provide insight into the biological process under investigation when the excitation conditions initiate the biologically optimized reaction pathway.^4^ The vital importance of restricting excitation conditions to the linear regime to ensure biological relevance has been discussed.^5^ In nature, photosystems highly optimized for absorbing light in the linear regime have even evolved non-photochemical quenching mechanisms to avoid the generation of deleterious excited states under high excitation.^6^ The existence of these mechanisms clearly demonstrates the need for the optimized biological process to be studied using relevant excitation conditions.

It has been shown that excessive excitation, as seen in all TR-SFX studies to date, leads to vastly different structural intermediates than what is biologically relevant,^7^ despite (in some cases) sharing a common final photoproduct.^8^ The magnitudes of the structural changes, correlation length of the motions, and specific pathways are critical to get right in order to understand structure–function relationships in biological systems. Even small differences in the magnitude of the structural changes, within typical resolution limits of time resolved crystallography, integrated over the large number of atoms involved in biological functions lead to significant errors in the barriers giving access to the conformational substates directing biological processes. Accordingly, given the large numbers of atoms typically involved (hundred to thousands) in these biologically active states, it is critical to initiate the process under biologically relevant conditions. The issue of excessive excitation in pump–probe experiments can no longer be ignored. Establishing well defined excitation limits for TR-SFX studies is essential to ensure their biological relevance.

In this work, the theoretical model used to identify the linear absorption regime uses time-dependent perturbation theory to identify the laser intensity at which the one-photon transition saturates. The onset of resonant multiphoton absorption (RMPA) beyond the saturation threshold is predicted here theoretically and observed experimentally.^8–11^ The linear excitation threshold is defined as the laser intensity for a given pulse duration, which, if exceeded, excites RMPA in the sample. RMPA occurs when multiple photons excite a single electron through a resonant intermediate state to higher electronic levels. For example, when studying a photoactive biological process that requires pumping the $S_{0}\to S_{1}$ transition near resonance, excessive pumping gives rise to the biologically irrelevant RMPA transition $S_{0}\to S_{1}\to S_{m}$, driving population beyond the biologically relevant target state and into higher energy states. This prepares an initial reaction state that is not well defined and involves excess thermal energy in the nonradiative relaxation process. As recently shown experimentally, the subsequent intermediate structures observed in TR-SFX studies do not then accurately reflect the biologically optimized reaction starting in the target state.^7^

## THEORETICAL FRAMEWORK

We consider the perturbation of a quantum system by a classical laser field to determine the vertical excitation dynamics. We take the laser pulse to be a sinusoidal electric field with frequency ω, turned on at t=0 and turned off at t=τ,

$$E(t)=\varepsilon e^{-i\omega t}+\varepsilon^{*}e^{i\omega t}.$$ (1)

Consider the eigenstates {|m⟩} that satisfy ${\hat{H}}_{0}|m\rangle =\hbar \omega_{m}|m\rangle$; we express the solution of the time-dependent Schrödinger equation,

$$i\hbar \frac{\partial \psi (r,t)}{\partial t}=\left({\hat{H}}_{0}+\hat{V}(t)\right)\psi (r,t),$$ (2)

as a linear combination of the basis states {|m⟩},

$$|\psi \rangle =\sum_{m}a_{m}(t)|m\rangle .$$ (3)

This ansatz leads to the set of coupled differential equations,

$$ih\frac{da_{m}}{dt}=\sum_{m\prime }a_{m\prime }(t)V_{mm\prime }e^{-i\omega_{m^{\prime }m}t},$$ (4)

where $\omega_{ij}=\omega_{i}-\omega_{j}$. We solve Eq. (4) using a perturbative approach.^12^ Applying time-dependent perturbation theory^13^ decouples the set differential equations allowing the population dynamics to be resolved to within the order of the interaction with the field,

$$\frac{da_{m}^{\left(N\right)}}{dt}={\left(i\hbar \right)}^{-1}\sum_{m\prime }a_{m^{\prime }}^{\left(N-1\right)}V_{mm\prime }e^{-i\omega_{m^{\prime }m}t},\;N=1,2,3\ldots .$$ (5)

The perturbation due to the laser field is given by

$$V_{ij}=-\mu_{ij}E(t),$$ (6)

where $\mu_{ij}$ is the dipole moment for the |i⟩→|j⟩ transition, related to the oscillator strength^14^ by

$$f_{ij}=\frac{2m_{e}\omega_{ij}}{3\hbar e^{2}}\mu_{ij}^{2}.$$ (7)

The first order amplitude found by setting N=1 in Eq. (5) and integrating to solve for $a_{m}(t)$ is given by

$$a_{m}^{\left(1\right)}(t)=\frac{\mu_{mg}\varepsilon }{\hbar (\omega_{mg}-\omega )}\left(e^{i(\omega_{mg}-\omega )t}-1\right).$$ (8)

This result makes use of the rotating wave approximation and assumes that population starts in the ground state |g⟩ at time t=0. Therefore, up to first order in the perturbation, the transition probability to an arbitrary excited state |m⟩ is given by the square of the amplitude in Eq. (8),

$$p_{m}^{\left(1\right)}(t)=\frac{{4|\mu_{mg}\varepsilon |}^{2}}{\hbar^{2}{\left(\omega_{mg}-\omega \right)}^{2}}{\text{sin}}^{2}\left(\frac{{(\omega }_{mg}-\omega )t}{2}\right).$$ (9)

Note the transition frequency $\omega_{mg}$ is perfectly defined in Eq. (9). This is of course not the case physically as there is an inherent linewidth associated with the transition. To account for this linewidth, we define an arbitrary line shape for the $\omega_{mg}$ transition, $\rho_{mg}(\omega_{mg})$. This arbitrary line shape is a normalized probability distribution that peaks at the mean transition frequency $\bar{\omega_{mg}}$. More generically, we define the lineshapes for any possible transition $\rho_{ij}(\omega_{ij}),\;i>j$ and ensure the normalization,

$$\int_{0}^{\infty }\rho_{ij}(\omega_{ij})d\omega_{ij}=1.$$ (10)

To implement the linewidth into the transition probability given in Eq. (9), we take the expected value of the transition probability with respect to the line shape distribution,

$$\left\langle p_{m}^{\left(1\right)}(t)\right\rangle =\int_{0}^{\infty }\rho_{mg}(\omega_{mg})\frac{{4|\mu_{mg}\varepsilon |}^{2}}{\hbar^{2}{\left(\omega_{mg}-\omega \right)}^{2}}{\text{sin}}^{2}\left(\frac{{(\omega }_{mg}-\omega )t}{2}\right)\;d\omega_{mg}.$$ (11)

The intensity of the laser is related to the electric field by

$$I=2n\epsilon_{0}c\varepsilon^{2},$$ (12)

allowing Eq. (11) to be written as

$$\left\langle p_{m}^{\left(1\right)}(t)\right\rangle =I\frac{\mu_{mg}^{2}}{2n\epsilon_{0}c\hbar^{2}}\int_{0}^{\infty }\rho_{mg}(\omega_{mg})\frac{{4\text{sin}}^{2}\left(\frac{{(\omega }_{mg}-\omega )t}{2}\right)}{{\left(\omega_{mg}-\omega \right)}^{2}}\;d\omega_{mg}.$$ (13)

Note the linear intensity dependence in Eq. (13); the linear absorption regime is characterized by the first order behavior as seen in Eq. (13).

The nonlinear absorption (multiphoton) regime is characterized by higher order interactions with the field. Setting N=2 in Eq. (5) gives the set of differential equations governing the second order interaction,

$$\frac{da_{m}^{\left(2\right)}}{dt}={-(i\hbar )}^{-1}\frac{\mu_{mm\prime }\mu_{m\prime g}\varepsilon^{2}}{\hbar (\omega_{m\prime g}-\omega )}\left(e^{i({\omega_{mm^{\prime }}+\omega }_{m^{\prime }g}-2\omega )t}-e^{i(\omega_{mm^{\prime }}-\omega )t}\right).$$ (14)

Here, the rotating wave approximation has been applied, and only the resonant two-photon absorption (RTPA) process, $\left|g\rangle \to |m^{\prime }\rangle \to |m\right\rangle$, is considered. We restrict the sum in Eq. (5) to a single resonant intermediate state, $\left|m^{\prime }\right\rangle$, corresponding to the target state for initiating the photoactive biological process. The transition probability to the higher lying electronic state |m⟩ due to second order interactions with the field is given by the square of amplitude $a_{m}^{\left(2\right)}(t),$ found by integrating Eq. (14),

$$p_{m}^{\left(2\right)}(t)={\left|\beta_{mm^{\prime }}^{\left(2\right)}\right|}^{2}f_{mm^{\prime }}^{\left(2\right)},$$ (15)

where $\beta_{mm\prime }^{\left(2\right)}$ carries the field and oscillator strength dependence,

$$\beta_{mm\prime }^{\left(2\right)}\equiv \frac{\mu_{mm^{\prime }}\mu_{m^{\prime }g}\varepsilon^{2}}{\hbar^{2}},$$ (16)

and $f_{mm\prime }^{\left(2\right)}$ carries the spectral and temporal dependence,

$$f_{mm\prime }^{\left(2\right)}(t,\;{\omega ,\;\omega }_{mm\prime },\;\omega_{m\prime g})\equiv {\frac{1}{{\left(\omega_{m^{\prime }g}-\omega \right)}^{2}}\left|\frac{e^{i({\omega_{mm^{\prime }}+\omega }_{m^{\prime }g}-2\omega )t}-1}{\left({\omega_{mm^{\prime }}+\omega }_{m^{\prime }g}-2\omega \right)}-\frac{e^{i(\omega_{{\mathrm{mm}}^{\prime }}-\omega )t}-1}{\left(\omega_{{\mathrm{mm}}^{\prime }}-\omega \right)}\right|}^{2}.$$ (17)

The linewidths of the transitions are incorporated by integrating over the relevant lineshapes,

$$\left\langle p_{m}^{\left(2\right)}(t)\right\rangle ={\left|\beta_{mm^{\prime }}^{\left(2\right)}\right|}^{2}\iint_{0}^{\infty }\rho_{mm\prime }(\omega_{mm\prime })\rho_{m\prime g}(\omega_{m\prime g})f_{mm^{\prime }}^{\left(2\right)}d\omega_{mm\prime }d\omega_{m\prime g}.$$ (18)

As expected, absorption driven by a second order interaction with the field goes quadratically with the laser intensity, as seen by the $\varepsilon^{4}$ dependence embedded in ${\left|\beta_{mm^{\prime }}^{\left(2\right)}\right|}^{2}$.

## LASER INTENSITY THRESHOLD

If excitation conditions drive sufficient population to |m⟩ through the target state $\left|m^{\prime }\right\rangle$, a biologically irrelevant reaction pathway is initiated starting in |m⟩. For example, the biological isomerization of the retinal chromophore in bacteriorhodopsin is initiated following $S_{0}\to S_{1}$ excitation. Isomerization of retinal initiated by the RTPA process $S_{0}\to S_{1}\to S_{4}$ is not biological. To ensure RTPA is prevented, the laser intensity used for photoexcitation must ensure that $\left\langle p_{m}^{\left(2\right)}(t)\right\rangle \ll 1$.

As laser intensity increases, saturation of the one-photon transition is followed by the onset of RTPA.^8^ The laser intensity threshold for RTPA is therefore defined as the saturation intensity for the one-photon transition. As the one-photon transition saturates, the linear intensity dependence seen in Eq. (13) breaks down as $\left\langle p_{m^{\prime }}^{\left(1\right)}(t)\right\rangle$ approaches unity. Treating the one-photon transition as a saturable absorber by replacing the linear intensity dependence in Eq. (13), $I\to \frac{I}{1+\frac{I}{I_{\text{sat}}}}$, the validity of the one-photon transition probability extends into the nonlinear regime in agreement with the intensity dependent study.^8^ This treatment ensures $\left\langle p_{m\prime }^{\left(1\right)}(t)\right\rangle$ is bounded below unity (as seen in Fig. 2). Setting Eq. (13) equal to unity and solving for the (pulse duration dependent) one-photon saturation intensity gives

$$I_{\text{sat}}=\frac{2n\epsilon_{0}c\hbar^{2}}{\mu_{m^{\prime }g}^{2}F_{m^{\prime }}^{\left(1\right)}(\tau ,\;\omega ,\;\rho_{m\prime g})},$$ (19)

where $F_{m}^{\left(1\right)}$ is the resonant enhancement factor for linear absorption defined by the maximum value of the overlap integral in Eq. (13) over the time duration of the pulse τ,

$$F_{m^{\prime }}^{\left(1\right)}(\tau ,\;\omega ,\;\rho_{m\prime g})\equiv {\text{max}\left(\int_{0}^{\infty }\rho_{m\prime g}\frac{{4\text{sin}}^{2}\left(\frac{{(\omega }_{m\prime g}-\omega )t}{2}\right)}{{\left(\omega_{m^{\prime }g}-\omega \right)}^{2}}\;d\omega_{m\prime g}\right)}_{t\in [0,\tau ]}.$$ (20)

Oscillations in the integral can occur for times $t>\frac{\pi }{\delta }$, where $\delta =\omega -\omega_{10}$ is the detuning from resonance. To ensure the accuracy of the threshold in Eq. (19) for pulse durations greater than π/δ, the maximum function is necessary.

Replacing the linear intensity dependence in Eq. (13) with that of a saturable absorber gives the transition probability to the target state $\left|m^{\prime }\right\rangle$, valid in the nonlinear regime,

$$\left\langle p_{m\prime }^{\left(1\right)}(t)\right\rangle =\frac{I}{1+\frac{I}{I_{\text{sat}}}}\cdot \frac{\mu_{m^{\prime }g}^{2}}{2n\epsilon_{0}c\hbar^{2}}F_{m^{\prime }}^{\left(1\right)}(t,\;\omega ,\;\rho_{m\prime g}),$$ (21)

where $I_{\text{sat}}$ is given by Eq. (19). We note that this transition probability does not correspond directly to the population of the excited states because the classical electric field used in the theoretical framework neglects to include effects such as spontaneous and stimulated emission, which are realized only by a quantized field.

## MODEL SYSTEM: BACTERIORHODOPSIN

A great deal of work has been done on bacteriorhodopsin (bR) both experimentally and theoretically with respect to its linear and nonlinear response.^10,15^ It is also a very important model system for understanding a whole class of biological processes including visual G-protein activation and pupillary reflex regulation in animals, ion pumping, ion-gating, and light-regulated enzymatic activity in eubacteria and archaea, central to living systems. The above-mentioned theoretical model was applied to bR, a light-driven proton pump found in the purple membrane of *Halobacterium salinarium.*^16^ Embedded within the protein is the retinal chromophore, which undergoes isomerization from the all-*trans* to 13-*cis* configuration upon photoexcitation to $S_{1}$. To test the theoretical framework, we applied the model to evaluate the intensity threshold in Eq. (19) as well as examined the transition probabilities in Eqs. (18) and (21). Intensity dependent population dynamics of bR have been studied in the high intensity regime, which provides a means of experimental comparison to the theoretical model. Applying the model to the singlet states of bR requires a high level quantum mechanics/molecular mechanics (QM/MM) calculation to obtain the vertical excitation energy and oscillator strengths of the relevant transitions, which are provided herein (see Table I).

**TABLE I.** Results of the 5r-SA-CASPT2 6-31G^*^ QM/MM model for bR. Oscillator strength f is related to the transition dipole moment by Eq. (7).

Singlet state	Vertical excitation energy (nm)	Oscillator strength, f (-)
$S_{1}$	562	$S_{0}\to S_{1}:0.94$
$S_{2}$	373	$S_{1}\to S_{2}:0.03$
$S_{3}$	326	$S_{1}\to S_{3}:0.27$
$S_{4}$	299	$S_{1}\to S_{4}:1.07$

**FIG. 1.**
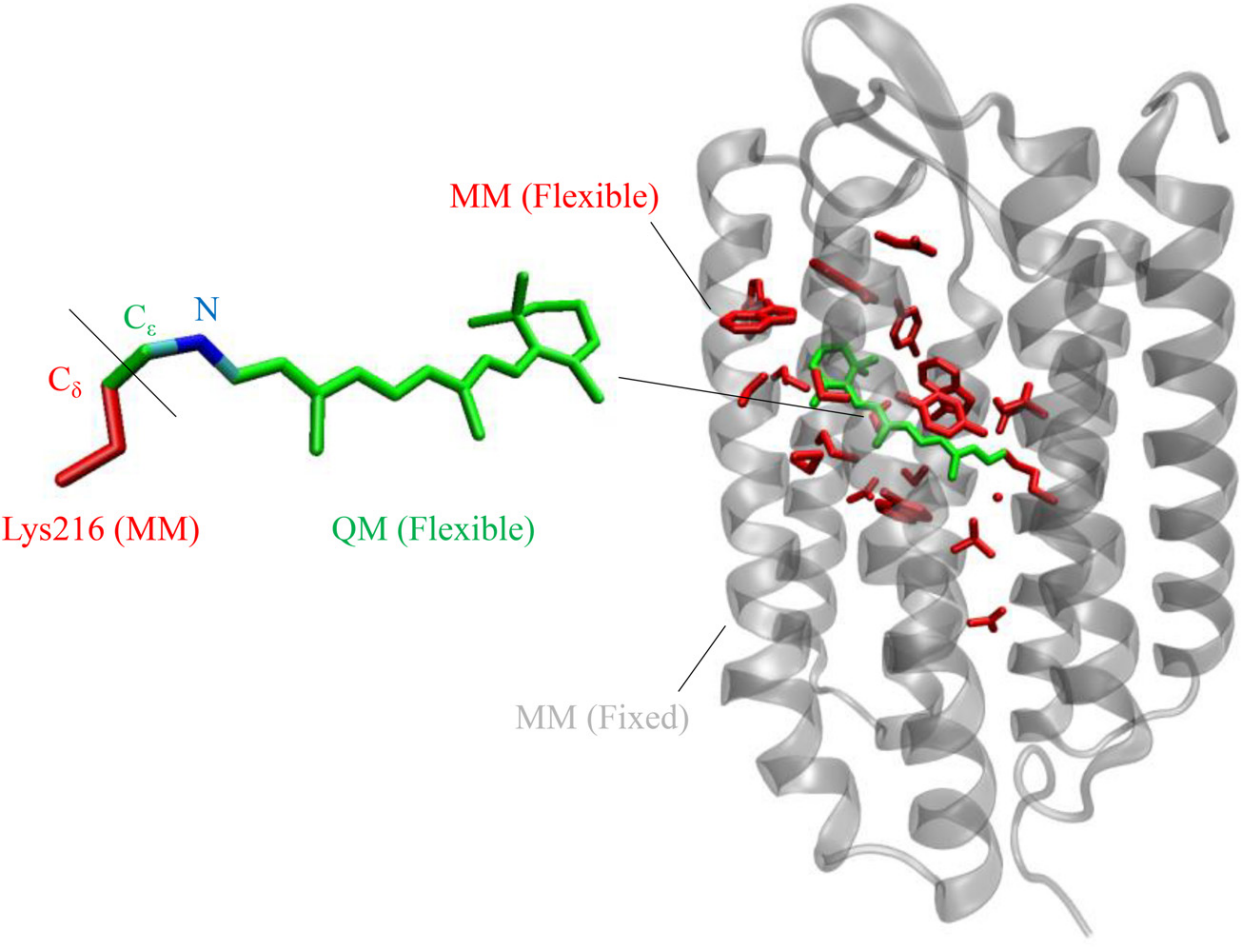
QM/MM model for bacteriorhodopsin. Generated using visual molecular dynamics.^23^

The QM/MM model for bR (see Fig. 1) was constructed using the 1.5 Å resolution crystallographic structure (Protein Data Bank ID: 6G7H)^17^ as described in previous work.^18^ The retinylidene chromophore, NH group, and C_ε_H_2_ atoms linked to the C_δ_ atom of the Lys216 form the QM layer, which is treated at the CASSCF/6-31G^*^ level of theory. The active space involves 12 electrons in 12 orbitals, which comprise the entire π system of the retinal chromophore moiety. The rest of the protein forms the MM layer, described by a modified AMBER94 force field^19^ with specific Lys216 side chain parameters.^20^ A H-link atom was introduced to saturate the bond between C_ε_ and C_δ_, which is also a part of the QM layer.

The ground state equilibrium geometry corresponding to this model was achieved through QM/MM optimization at the 2-root state average CASSCF/6-31G^*^ level. During the optimization, the QM layer, all side-chains, and water molecules within a cavity defined by the model construction protocol were allowed to remain flexible, while all other atoms were kept frozen. Based on the ground state equilibrium geometry, the vertical excitation energy and corresponding oscillator strength were evaluated at the 5-root state average CASPT2/6-31G^*^ level. All calculations were performed using the Molcas/Tinker package.^21,22^

The line shape functions of the singlet state bR transitions are assumed to be Lorentzian,

$$\rho_{ij}(\omega_{ij})=\frac{\Gamma_{ij}}{2\pi }\;\left(\frac{1}{{\left({\bar{\omega }}_{ij}-\omega_{ij}\right)}^{2}+{\left(\frac{\Gamma_{ij}}{2}\right)}^{2}}\right),$$ (22)

where the FWHM of the linear transition is $\Gamma_{10}=\frac{2\pi }{26fs},$ equivalent to $1.28\times 10^{3}$ cm^−1^. The dephasing time of 26 fs was calculated from the 2DPE bR study.^24^ The general results and trends will not be affected if a Gaussian or inhomogeneous line shape was used instead as they would all conform to the observed absorption spectrum and transition probabilities for the first excited state. Additionally, only the linewidth of the linear transition is required to evaluate the intensity threshold in Eq. (19).

## RESULTS AND DISCUSSION

**FIG. 2.**
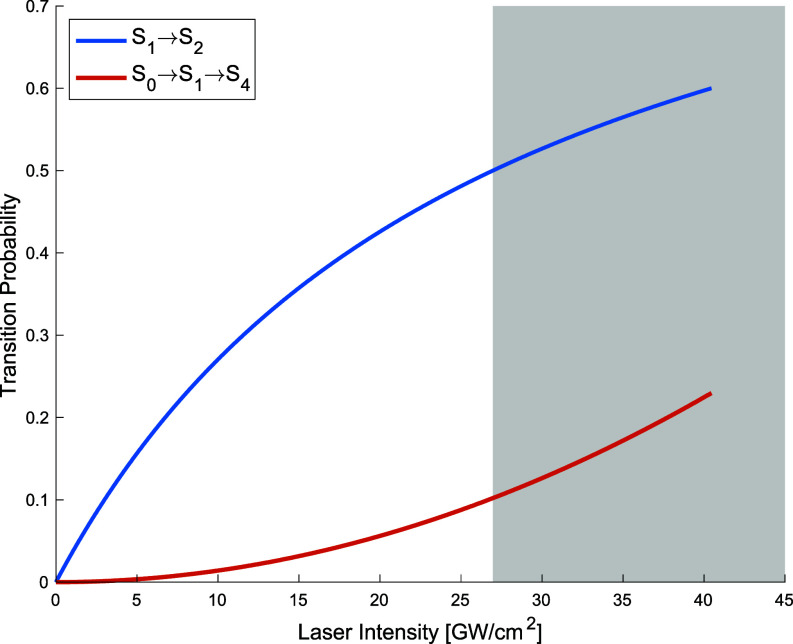
Vertical excitation dynamics of bacteriorhodopsin pumped at 570 nm for 25 fs. Saturation of one-photon transition probability (blue) followed by onset of substantial two-photon transition probability (red) calculated using Eqs. (21) and (18), respectively. Shaded region indicates nonlinear absorption regime, the onset of which is calculated using Eq. (19). The dominant RTPA process in bR pumped at 570 nm is the $S_{0}\to S_{1}\to S_{4}$ transition. This is due to the relatively large oscillator strength $S_{1}\to S_{4}$ (see Table I). For visual clarity, only this dominant transition is displayed. The dephasing time of the $S_{1}\to S_{4}$ transition was taken to be 75% of the $S_{0}\to S_{1}$ dephasing time corresponding to a linewidth of $1.71\times 10^{3}$ cm^−1^.

To demonstrate the effect of resonant two-photon absorption (RTPA) on the transition probability to higher lying states, Fig. 2 shows saturation of the one-photon transition ( $S_{0}\to S_{1}$), followed by the onset of RTPA ( $S_{0}\to S_{1}\to S_{4}$) driving population into $S_{4}$. As predicted theoretically in Fig. 2 and experimentally observed in Ref. 8, saturation of the one-photon transition and the onset of substantial RTPA occurs beyond the intensity threshold defined by Eq. (19).

Figure 2 replicates the excitation conditions (570 nm, 25 fs) used in the intensity dependent transient absorption study of bR.^8^ For a square pulse, the linear saturation intensity predicted by Eq. (19) for these pulse parameters corresponds to a fluence of about $2\times 10^{15}$ photons/cm^2^. This agrees with the experimentally estimated saturation level of $4.8\times 10^{15}$ photons/cm^2^ seen with a Fourier-transform-limited pulse.^8^

The onset of nonlinear absorption in bR pumped at 535 nm for pulse durations on the order of a picosecond was observed to be about 30 GW/cm^2^.^9^ Evaluating Eq. (19) with these pulse parameters theoretically estimates the onset of nonlinear absorption to occur beyond 15 GW/cm^2^. The agreement between the theoretical model and the observed behavior^8,9^ clearly demonstrates from first principles the effect that excessive excitation has on multiphoton absorption.

## CONCLUSION

Employing excitation conditions in TR-SFX studies that exceed the threshold in Eq. (19) leads to vastly different processes than which is biologically relevant.^7^ For bR and many other light absorbing proteins, there have been multiple instances of TR-SFX studies employing excitation conditions beyond the linear threshold.^5^ For example, the study of retinal isomerization in bR conducted by Nogly *et al.* in 2018 employed excitation conditions with a peak laser intensity^5^ of 4800 GW/cm^2^; the intensity threshold calculated using Eq. (19) is two orders of magnitude lower at about 15 GW/cm^2^ (laser detuning 33 nm and pulse duration 100 fs). As seen in Fig. 2, this high level of excitation leads to RMPA driving significant population, even predominantly, into higher energy states, which brings to question the biological relevance of the observed structural changes. As seen in Ref. 8, the production of the 13-*cis* photoproduct at intensities beyond the threshold does not occur via the optimized biological reaction pathway beginning with the I_460_ state. Therefore, the reaction probed by Nogly *et al.* is the biologically irrelevant and experimentally observed^10,15^ production of the 13-*cis* photoproduct starting at a higher energy state excited by RMPA.

There may be more factors to consider when selecting excitation conditions for the study of structural dynamics. These include nonresonant multiphoton absorption channels involving amino acid transitions in near resonance conditions. The excitation conditions have been so high that avalanche ionization and plasma formation in the irradiated samples could be contributing to the observed structural changes. Even the internal waters stabilizing the protein matrix, that do not absorb significantly till the far UV (< 200 nm), would experience significant multiphoton ionization at typical excitation intensities used. Peak powers, sufficient to generate seed electrons capable of initiating avalanche ionization, occur at intensities as low as 100 GW/cm^2^ for nanosecond pulses,^25^ and 1000 GW/cm^2^ for fs excitation pulses.^26^ These processes will occur at significantly lower peak powers for the near resonant conditions for amino acids that form the bulk of the protein matrix. It is important to keep the excitation conditions well below these regimes to access biologically relevant structural changes.

The onset of biologically irrelevant multiphoton absorption clearly occurs at excitation levels that exceed the linear threshold given by Eq. (19). It is essential in the study of structural dynamics that excitation conditions be selected in the linear regime to avoid RMPA from affecting the observed dynamics. This statement refers to all time resolved studies of structural dynamics involved in chemical and biological processes that require well defined initial states to properly connect structural changes to the process of interest. For biological processes in particular, nature has necessarily optimized light activated biological processes in the 1-photon or weak excitation regime corresponding to solar fluences. The importance of implementing a standard protocol for selecting excitation conditions corresponding to this biologically relevant regime cannot be overlooked. The theoretical framework provided here can now be used to follow the proposed TR-SFX excitation guidelines.^5^ Essentially, this work provides the operating limits to prepare well defined initial states for initiating biologically relevant structural changes in order to properly capture the fundamental connection between structure and dynamics in understanding biological processes.

## Data Availability

Data sharing is not applicable to this article as no new data were created or analyzed in this study.

## References

[1] M. Schmidt , in *Protein Crystallography*, edited by A. Wlodawer , Z. Dauter , and M. Jaskolski ( Springer, New York, 2017), pp. 273–294.

[2] G. Brändén and R. Neutze , “ Advances and challenges in time-resolved macromolecular crystallography,” Science 373(6558), eaba0954 (2021). 10.1126/science.aba0954 34446579

[3] R. J. D. Miller , “ Femtosecond crystallography with ultrabright electrons and x-rays: Capturing chemistry in action,” Science 343(6175), 1108–1116 (2014). 10.1126/science.1248488 24604195

[4] R. J. D. Miller , O. Paré-Labrosse , A. Sarracini , and J. E. Besaw , “ Three-dimensional view of ultrafast dynamics in photoexcited bacteriorhodopsin in the multiphoton regime and biological relevance,” Nat. Commun. 11(1), 1240 (2020). 10.1038/s41467-020-14971-0 32144255 PMC7060340

[5] J. E. Besaw and R. J. D. Miller , “ Addressing high excitation conditions in time-resolved x-ray diffraction experiments and issues of biological relevance,” Curr. Opin. Struct. Biol. 81, 102624 (2023). 10.1016/j.sbi.2023.102624 37331203

[6] P. Müller , X.-P. Li , and K. K. Niyogi , “ Non-photochemical quenching. A response to excess light energy,” Plant Physiol. 125(4), 1558–1566 (2001). 10.1104/pp.125.4.1558 11299337 PMC1539381

[7] T. R. M. Barends, A. Gorel, S. Bhattacharyya, G. Schirò, C. Bacellar, C. Cirelli, J.-P. Colletier, L. Foucar, M. L. Grünbein, E. Hartmann, M. Hilpert, J. M. Holton, P.J.M. Johnson, M. Kloos, G. Knopp, B. Marekha, K. Nass, G. Nass Kovacs, D. Ozerov, M. Stricker, M. Weik, R.B. Doak, R.L. Shoeman, C.J. Milne, M. Huix-Rotllant, M. Cammarata, and I. Schlichting, “Influence of pump laser fluence on ultrafast myoglobin structural dynamics,” Nature, 2024, 1–7. 10.1038/s41586-024-07032-9 PMC10881388 38355794

[8] A. C. Florean , D. Cardoza , J. L. White , J. K. Lanyi , R. J. Sension , and P. H. Bucksbaum , “ Control of retinal isomerization in bacteriorhodopsin in the high-intensity regime,” Proc. Natl. Acad. Sci. U. S. A. 106(27), 10896–10900 (2009). 10.1073/pnas.0904589106 19564608 PMC2708765

[9] G. Nass Kovacs , J.-P. Colletier , M. L. Grünbein , Y. Yang , T. Stensitzki , A. Batyuk , S. Carbajo , R. B. Doak , D. Ehrenberg , L. Foucar , R. Gasper , A. Gorel , M. Hilpert , M. Kloos , J. E. Koglin , J. Reinstein , C. M. Roome , R. Schlesinger , M. Seaberg , R. L. Shoeman , M. Stricker , S. Boutet , S. Haacke , J. Heberle , K. Heyne , T. Domratcheva , T. R. M. Barends , and I. Schlichting , “ Three-dimensional view of ultrafast dynamics in photoexcited bacteriorhodopsin,” Nat. Commun. 10(1), 3177 (2019). 10.1038/s41467-019-10758-0 31320619 PMC6639342

[10] T. Fischer and N. A. Hampp , “ Two-photon absorption of bacteriorhodopsin: Formation of a red-shifted thermally stable photoproduct F_620_,” Biophys. J. 89(2), 1175–1182 (2005). 10.1529/biophysj.104.055806 15894635 PMC1366602

[11] R. J. D. Miller , V. I. Prokhorenko , A. M. Nagy , S. A. Waschuk , L. S. Brown , and R. R. Birge , “ Coherent control of retinal isomerization in bacteriorhodopsin,” Science 313(5791), 1257–1261 (2006). 10.1126/science.1130747 16946063

[12] R. W. Boyd , *Nonlinear Optics*, 4th ed. ( Elsevier, 2020).

[13] J. J. Sakurai , *Modern Quantum Mechanics*, 3rd ed. ( Cambridge University Press, Cambridge, 2021).

[14] R. C. Hilborn , “ Einstein coefficients, cross sections, *f* values, dipole moments, and all that,” Am. J. Phys. 50(11), 982–986 (1982). 10.1119/1.12937

[15] X. Yu , B. Yao , M. Lei , P. Gao , and B. Ma , “ Femtosecond laser-induced permanent anisotropy in bacteriorhodopsin films and applications in optical data storage,” J. Mod. Opt. 60(4), 309–314 (2013). 10.1080/09500340.2013.774067

[16] J. M. Kim , P. J. Booth , S. J. Allen , and H. G. Khorana , “ Structure and function in bacteriorhodopsin: The role of the interhelical loops in the folding and stability of bacteriorhodopsin,” J. Mol. Biol. 308(2), 409–422 (2001). 10.1006/jmbi.2001.4603 11327776

[17] P. Nogly , T. Weinert , D. James , S. Carbajo , D. Ozerov , I. Schapiro , G. Schertler , R. Neutze , and J. Standfuss , “ Retinal isomerization in bacteriorhodopsin captured by a femtosecond x-ray laser,” Acta Crystallogr., Sect. A 74(a2), e171 (2018). 10.1107/S2053273318092689 29903883

[18] L. Pedraza-González , M. del C. Marín , L. De Vico , X. Yang , and M. Olivucci , “ On the automatic construction of QM/MM models for biological photoreceptors: Rhodopsins as model systems,” in *Challenges and Advances in Computational Chemistry and Physics* ( Springer, 2021), Vol. 31, pp. 1–75.

[19] W. D. Cornell , P. Cieplak , C. I. Bayly , I. R. Gould , K. M. Merz , D. M. Ferguson , D. C. Spellmeyer , T. Fox , J. W. Caldwell , and P. A. Kollman , “ A second generation force field for the simulation of proteins, nucleic acids, and organic molecules *J. Am. Chem. Soc.* 1995, *117*, 5179–5197,” J. Am. Chem. Soc. 118(9), 2309–2309 (1996). 10.1021/ja955032e

[20] N. Ferré , A. Cembran , M. Garavelli , and M. Olivucci , “ Complete-active-space self-consistent-field/Amber parameterization of the Lys296-retinal-Glu113 rhodopsin chromophore-counterion system,” Theor. Chem. Acc. 112(4), 335–341 (2004). 10.1007/s00214-004-0593-0

[21] F. Aquilante , J. Autschbach , R. K. Carlson , L. F. Chibotaru , M. G. Delcey , L. De Vico , I. Fdez. Galván , N. Ferré , L. M. Frutos , L. Gagliardi , M. Garavelli , A. Giussani , C. E. Hoyer , G. Li Manni , H. Lischka , D. Ma , P. Å. Malmqvist , T. Müller , A. Nenov , M. Olivucci , T. B. Pedersen , D. Peng , F. Plasser , B. Pritchard , M. Reiher , I. Rivalta , I. Schapiro , J. Segarra-Martí , M. Stenrup , D. G. Truhlar , L. Ungur , A. Valentini , S. Vancoillie , V. Veryazov , V. P. Vysotskiy , O. Weingart , F. Zapata , and R. Lindh , “ Molcas 8: New capabilities for multiconfigurational quantum chemical calculations across the periodic table,” J. Comput. Chem. 37(5), 506–541 (2016). 10.1002/jcc.24221 26561362

[22] J. A. Rackers , Z. Wang , C. Lu , M. L. Laury , L. Lagardère , M. J. Schnieders , J. P. Piquemal , P. Ren , and J. W. Ponder , “ Tinker 8: Software tools for molecular design,” J. Chem. Theory Comput. 14(10), 5273 (2018). 10.1021/acs.jctc.8b00529 30176213 PMC6335969

[23] W. Humphrey , A. Dalke , and K. Schulten , “ VMD: Visual molecular dynamics,” J. Mol. Graphics 14(1), 33–38 (1996). 10.1016/0263-7855(96)00018-5 8744570

[24] S. Gozem , P. J. M. Johnson , A. Halpin , H. L. Luk , T. Morizumi , V. I. Prokhorenko , O. P. Ernst , M. Olivucci , and R. J. D. Miller , “ Excited-state vibronic dynamics of bacteriorhodopsin from two-dimensional electronic photon echo spectroscopy and multiconfigurational quantum chemistry,” J. Phys. Chem. Lett. 11(10), 3889–3896 (2020). 10.1021/acs.jpclett.0c01063 32330041 PMC9198827

[25] N. Linz , S. Freidank , X.-X. Liang , H. Vogelmann , T. Trickl , and A. Vogel , “ Wavelength dependence of nanosecond infrared laser-induced breakdown in water: Evidence for multiphoton initiation via an intermediate state,” Phys. Rev. B 91(13), 134114 (2015). 10.1103/PhysRevB.91.134114

[26] N. Linz , S. Freidank , X.-X. Liang , and A. Vogel , “ Wavelength dependence of femtosecond laser-induced breakdown in water and implications for laser surgery,” Phys. Rev. B 94(2), 024113 (2016). 10.1103/PhysRevB.94.024113

